# Nuclear wastewater decontamination by 3D-Printed hierarchical zeolite monoliths†

**DOI:** 10.1039/c9ra09967k

**Published:** 2020-02-05

**Authors:** Oded Halevi, Tzu-Yu Chen, Pooi See Lee, Shlomo Magdassi, Joseph A. Hriljac

**Affiliations:** School of Chemistry, University of Birmingham Edgbaston Birmingham B15 2TT UK; Diamond Light Source Ltd, Harwell Science and Innovation Campus Didcot OX11 0DE UK joseph.hriljac@diamond.ac.uk; Materials and Engineering Research Institute, Faculty of Science, Technology and Arts, Sheffield Hallam University City Campus, Howard Street Sheffield S1 1WB UK; CREATE NTU-HUJ Programme Enterprise Wing 138602 Singapore; Casali Center for Applied Chemistry, Institute of Chemistry, The Hebrew University of Jerusalem Jerusalem 91904 Israel magdassi@mail.huji.ac.il; School of Materials Science and Engineering, Nanyang Technological University 639798 Singapore pslee@ntu.edu.sg

## Abstract

The selective removal of radioactive cationic species, specifically ^137^Cs^+^ and ^90^Sr^2+^, from contaminated water is critical for nuclear waste remediation processes and environmental cleanup after accidents, such as the Fukushima Daiichi Nuclear Power Plant disaster in 2011. Nanoporous silicates, such as zeolites, are most commonly used for this process but in addition to acting as selective ion exchange media must also be deployable in a correct physical form for flow columns. Herein, Digital Light Processing (DLP) three-dimensional (3D) printing was utilized to form monoliths from zeolite ion exchange powders that are known to be good for nuclear wastewater treatment. The monoliths comprise 3D porous structures that will selectively remove radionuclides in an engineered form that can be tailored to various sizes and shapes as required for any column system and can even be made with fine-grained powders unsuitable for normal gravity flow column use. 3D-printed monoliths of zeolites chabazite and 4A were made, characterized, and evaluated for their ion exchange capacities for cesium and strontium under static conditions. The 3D-printed monoliths with 50 wt% zeolite loadings exhibit Cs and Sr uptake with an equivalent ion-capacity as their pristine powders. These monoliths retain their porosity, shape and mechanical integrity in aqueous media, providing a great potential for use to not only remove radionuclides from nuclear wastewater, but more widely in other aqueous separation-based applications and processes.

## Abstract 2

3D-printed monoliths of zeolites chabazite and 4A were made, characterized, and shown effective for removing strontium and caesium from water.

## Graphical abstract

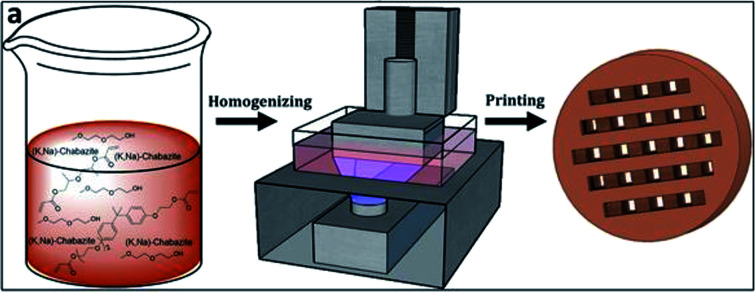

## 1. Introduction

Nuclear power plants generate about 11% of world electricity today.^1^ Although nuclear fission is environmentally benign in the sense of not producing any carbon emissions, it is of course necessary to deal with the nuclear waste that is produced and be able to rapidly respond to and mitigate the effects of accidental releases of radionuclides to the environment such as the disasters that occurred at Chernobyl in 1986 and the Fukushima Daiichi plant in 2011. The source for the majority of the medium-lived radiation in spent fuels are two radionuclides, ^137^Cs and ^90^Sr, which both have high fission yields and half-lives of around 30 years. As the two radionuclides form many soluble salts, they are most likely to contaminate water bodies. In addition, given the relatively high volatility of cesium salts, it is the species that spreads most widely in the environment after accidental releases. For example, the Fukushima accident released approximately 10 PBq of ^137^Cs into environment,^2^ the removal of this radionuclide continues to be a significant part of cleanup.

For more than 40 years, aluminosilicate zeolites have been playing an important role as ion exchange media for nuclear waste treatment by selective removal of cesium and strontium from wastewater. In 1985 British Nuclear Fuels Limited (BNFL) successfully commissioned the Site Ion Exchange Effluent Plant (SIXEP) at Sellafield, which uses a naturally occurring zeolite, clinoptilolite, to remove cesium and strontium from all water bodies before discharge into the sea.^3^ This led to a dramatic decrease in the contamination of effluent. Two other zeolites which show good selectivity for Cs^+^ and Sr^2+^ are chabazite and zeolite 4A. Chabazite is found naturally as a sodium-rich form (herschelite) and shows good selectivity for Cs^+^ and moderate selectivity for Sr^2+^.^4,5^ Dyer and Zubair have shown that the selectivity is thermodynamically favorable for many cations (Na^+^, K^+^, Rb^+^, Mg^2+^, Ca^2+^, Sr^2+^ and Ba^2+^) and generally correlates with the size difference between Cs^+^ and the replaceable cation.^6^ It has been widely used in clean-up efforts at Three Mile Island, Chernobyl and Fukushima. Synthetic zeolite A in the sodium form (commercially known as zeolite 4A), has a polyhedral open-cage structure with pores roughly 4 Å in size and most of the cation sites are accessible. The sodium ions can be fully exchanged, showing better selectivity towards smaller metal cations, such as Sr^2+^.^7^ The selectivity of Sr^2+^ over Na^+^ or K^+^ has been attributed to the high framework charge and, hence, strong electrostatic attraction of the divalent cation to the framework.^8^ Nuclear waste treatment can be demanding, in some cases the radionuclides must be removed from highly radioactive solutions that are also extremely acidic or caustic, where natural zeolites suffer due to their nature as aluminosilicates. Various synthetic materials such as titano-, zircono-silicates^9–11^ or metal oxides^12,13^ have been developed and proved more useful in these cases.

Synthetic ion exchangers can be produced with a wide variety of tailored chemical properties and ion exchange selectivity in the laboratory, but certain physical and mechanical characteristics may limit their applicability and efficiency for actual use. Therefore, most candidate materials do not make it from the bench into a plant. One of those limitations is when the material is only available as a fine-grained loose powder, such as many microporous solids that are produced with a particle size from sub-micron to a few tens of microns. Although they have a high surface area, which is beneficial for fast ion exchange, they might be packed very tightly in columns which can lead to reduced flow rates and ultimately leading to a flow blockage.^14^ They are also more difficult to handle and can form radioactive dusts during dry handling and disposal after use. It is for this reason that the naturally sourced aggregate SIXEP clinoptilolite is crushed and sieved so that only particles between 0.4 and 0.8 mm are put into the columns.^15^ For fine-grained synthetic materials this is not an option, so binder materials must be used to produce beads embedded with the zeolite to retain good mechanical strength. For example IONSIV® R9120-B is a commercial crystalline silicotitanate (CST) powder produced in bead form using the inorganic binder Zr(OH)_4_,^16,17^ which is compatible with potential final waste forms and processes such as vitrification.

In this report, we present a new approach that provides a breakthrough solution to the above problems, based on the fabrication of ion exchanger monoliths by three-dimensional (3D) printing. 3D-printing is an emerging technology, offering various methods for fabrication of objects of varying size, shape and complexity. The common denominator of all 3D-printing methods is the layer by layer deposition of materials, to form a 3D solid object.^18^ This printing technology is being utilized for manufacturing and prototyping in many fields such as construction,^19^ microfluidics,^20^ and soft robotics.^21^ It is also used to form complex structures of polymers, embedded with functional materials, thus bringing additional functionalities to the printed structures. Such functional 3D-printing has been demonstrated for example with graphene and carbon nanotubes to form conductive objects,^22^ ceramic nanoparticles for piezoelectric applications,^23^ and metal–organic frameworks to form gas adsorbing structures.^24^ Monolithic zeolite-containing structures have been produced and used in dry applications such as gas adsorption,^25^ gas separation,^26,27^ and catalytic cracking.^28,29^ Recently, a 3D printed metakaolin geopolymer was reported that withstood aqueous ion exchange, this was then used as a precursor to a ceramic monolith with a designed shape.^30^

To date, no 3D-printed zeolite monoliths have been produced specifically for ion exchange of aqueous media where they would need to be both insoluble and stable with regards shape retention over time when exposed to water.^31^ Herein, the digital light processing (DLP) method was utilized,^32^ which is based on UV-polymerization of each layer to form the 3D-printed structure. This enables excellent control over the porosity and the physical and chemical properties of the polymeric matrix, making it possible to tailor it according to the specific application requirements. Furthermore, the binder in this report is an organic polymer, in contrary to the inorganic bentonite clay reported previously, which is not suitable for liquid column flow operation, due to the physical deformation under the flow of liquid.^33^ In addition, the commonly used bentonite binder possesses cation-exchange capacity.^34–36^ Therefore, when it is used as a binder for screening new ion exchangers in the laboratory scale, it interferes with the screening of the retention and selectivity properties of the selected materials of interests.

The photopolymerizable monomers were mixed with the zeolite powder and were locally polymerized by ultra-violet (UV) light during printing in the presence of a porogenic solvent. The variety of available photopolymerizable monomers enables tailoring of the binder's physical and chemical properties such as stretchability,^37^ temperature responsivity,^38^ and hydrophobicity.^39^ The use of the porogenic solvent enables formation of a porous 3D structures,^40^ which is a crucial factor for maintaining the accessibility and functionality of the zeolite powder in the composite. The 3D monoliths have a hierarchical structure so that they are readily useable for liquid flow separation experiments, due to the design of cross-channels along the cylinder radius that connects the channels running along the cylinder length. Two zeolites, synthetic chabazite and commercial zeolite 4A, were printed and the hierarchical monoliths were evaluated to establish that the zeolites remain intact and retain the ability to remove Cs and Sr from aqueous solution. Depending upon the choice of ion exchanger, the target ions for removal do not have to be radionuclides, hence this technology could be readily adapted to removal of other cations such as environmentally damaging heavy metals or ammonia.

## 2. Results and discussion

### 2.1 Formulation preparation and 3D-printing

**Fig. 1:**
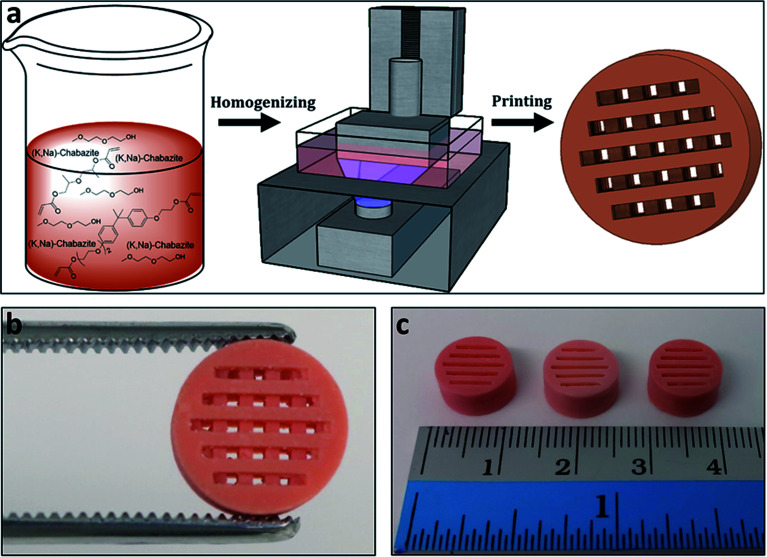
(a) Schematic overview of the printing process; first a dispersion of the zeolite was formed within the polymerizable monomers and porogenic solvent, then the formulation was 3D-printed by the DLP method. (b and c) The printed zeolite-embedded monolithic structures.

Polymeric cylindrical monolithic nets, embedded with chabazite (3D-CHA) or zeolite 4A (3D-4A), have been printed using the DLP method (Fig. 1a). For optimal performance of the zeolite in an ion-exchange column configuration, the printed structure should enable the flow of the solution through the column, and the polymeric matrix should allow the access of the cations from the feed to the surface of the zeolite particles, so that they can exchange into the pores of the embedded zeolite. Therefore, each object was printed as a porous cylindrical net, as seen in Fig. 1b and c. The cylinders can be stacked to the required height providing adaptability in practice.

**Fig. 2:**
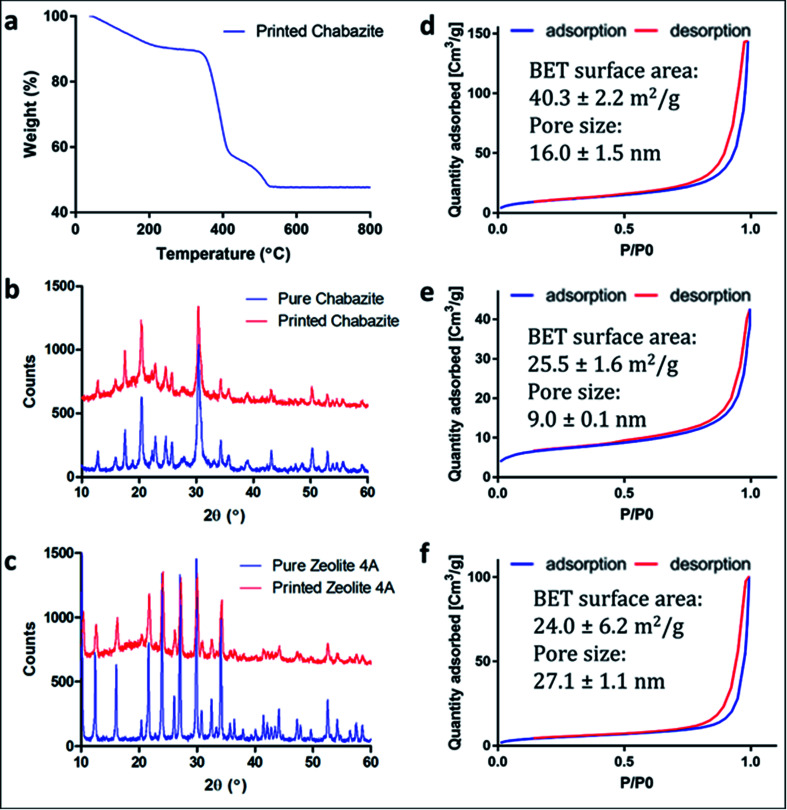
(a) TGA curve of 3D-CHA. (b and c) Comparison between the PXRD of zeolite powders and the zeolite embedded printed structures (b) 3D-CHA and pure chabazite powder; (c) 3D-4A and pure zeolite 4A powder. The patterns of the printed systems have been offset for clarity. (d–f) N_2_ adsorption isotherms of (d) 3D-CHA; (e) the pure chabazite powder; (f) the printed polymer.

To enable maximal accessibility and prevent the polymer from blocking the zeolite active sites, a high surface area of the polymer was required. As reported earlier, the overall porosity of the polymer can be controlled mainly by tailoring the fraction of the porogenic solvent and the concentration of the cross-linker monomer.^40,41^ The porogenic solvent causes low swelling of the formed polymer with the monomers during the polymerization process. This leads to a later-stage phase separation, which results in smaller pores and a higher surface area. Diethylene glycol monomethyl ether (DM), which is a good solvent for the selected monomers, was chosen as the porogenic solvent. The concentration of the cross-linker should affect the pores size as well. A higher cross-linker concentration would lead to a higher surface area.^42^ Consequently, bifunctional acrylate monomers were chosen: ethoxylated (3) bisphenol A dicarylate (SR-349) as the main monomer, and dipropylene glycol diacrylate (SR-508) that was added to decrease the viscosity of the formulation, to enable printing at high quality. Since the printing is based on photopolymerization, the printing composition contained a photoinitiator, diphenyl(2,4,6-trimethylbenzoyl)phosphine oxide (TPO), as well as a pigment (Orasol orange 272), to obtain a high printing resolution. The weight concentration of the zeolite in the starting formulation was 25 wt% to yield solid printed structures with 45–50 wt% zeolite. Thermal gravimetric analysis (Fig. 2a) of a 3D-CHA sample showed several stages of weight loss upon heating, due to dehydration of the zeolite and decomposition of the polymer, giving a final 47.7% remaining weight after heating above 600 °C.

### 2.2 Structural characterization of the printed models

Powder X-ray diffraction (PXRD) measurements of the original zeolite powders and portions of crushed 3D monoliths verified that the zeolite crystal structures were not altered during the fabrication process (Fig. 2b and c). The higher backgrounds from *ca.* 10–30° for the printed systems are due to the presence of the amorphous polymeric phase. A minor decrease in crystallinity of the zeolites, indicated by peak broadening, was also observed.

Fig. 2d–f show the nitrogen adsorption isotherms of printed samples degassed at 70 °C (we did not apply higher temperature in order to avoid polymer deformation). The low BET surface area in the chabazite sample results from blockage of the channels for N_2_ at 77 K by the solvent, and the incomplete clearance in the pores at such low degassing temperature. This claim will be confirmed later in this paper, by ion-exchange tests in solutions. All of the three samples show similarity in the shape of isotherms for the monoliths and the pristine powder, as well as H3-type hysteresis loops, which indicate the mesoporous nature of the 3D-printed monoliths. The microporous structure could not be identified due to the low degassing temperature.

### 2.3 Morphologies of the printed models

**Fig. 3:**
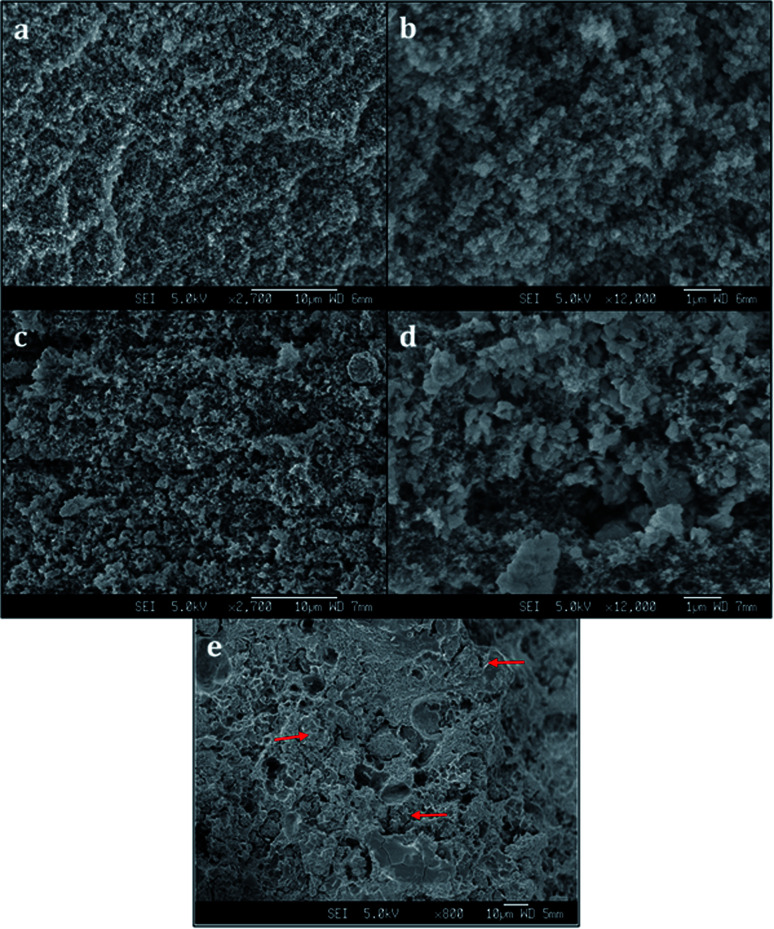
SEM images of (a and b) 3D-printed polymer, (c and d) 3D-CHA monolith, (e) 3D-4A monolith.

Scanning electron microscopy (SEM) images reveal a porous structure in the printed polymer (Fig. 3a and b). The voids are uniform and sized in the sub-micrometer scale. The SEM image of the chabazite-embedded monolith (Fig. 3c and d) shows that the chabazite grains are distributed evenly within the porous polymeric matrix. The zeolite 3D-4A (Fig. 3e) was printed with the same composition of polymer. The microcrystals of zeolite 4A, indicated with arrows, were well embedded in the polymer matrix. However, it shows a much denser morphology compared to 3D-CHA. The texture is less fluffy, and more agglomerates were observed.

### 2.4 Ion exchange tests

In order to verify the removal of Cs or Sr and investigate the mechanical integrity, the printed objects were tested for ion exchange and characterized using SEM-EDX, Infinite Focus Microscopy (IFM), XRD and X-ray Fluorescence (XRF) spectroscopy.

**Fig. 4:**
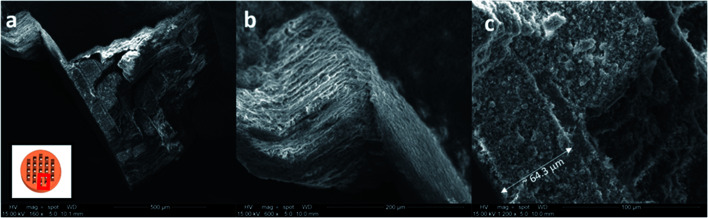
SEM images of Cs-exchanged 3D-printed monolith (a) an overview (b) side view (c) top view of the rod taken from the grid.

Fig. 4 shows the morphologies of Cs-exchanged 3D-CHA printed monolith after shaking in aqueous solution for 24 hours. A rod was taken from the grid for SEM observation as shown in the inset of Fig. 4a. This demonstrates that the printed structures are retained intact after ion exchange and the porous texture of each layer is consistent. Chabazite is uniformly blended into the polymer matrix and the rough surfaces of all sides of the rod and each porous layer allow the aqueous media to access through the pellet, as shown in Fig. 4b and c. It also demonstrates the excellent resolution of the DLP technique (Fig. 4c) enabling 3D printing to fabricate an object detailed to ∼60 μm.

**Fig. 5:**
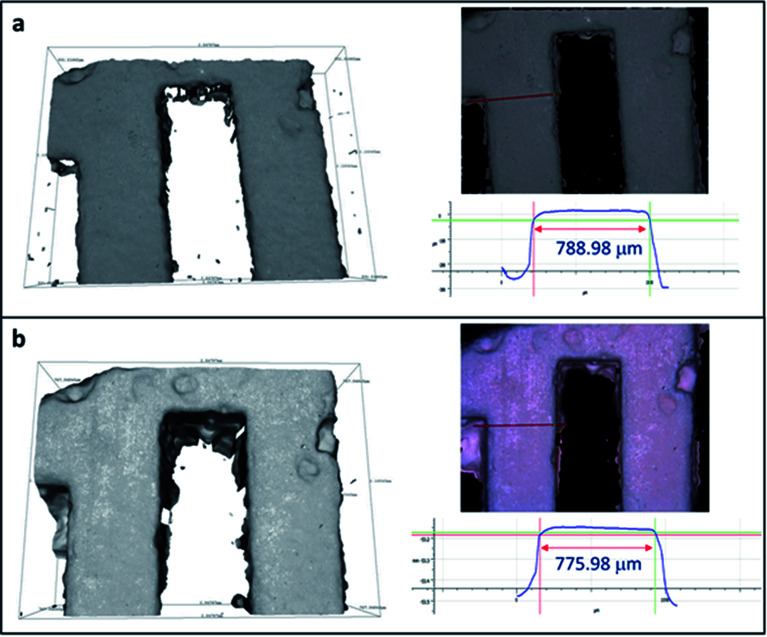
IFM images of 3D-printed monolith (a) before and (b) after Cs ion exchange and their profile measurements.

3D printed monoliths were subjected to IFM for investigating the robustness of the monoliths after being shaking in water media. Since the ion exchangers used in the nuclear industry will be immobilized rather than regenerated and reused to avoid risk of spreading the hazards, the integrity investigation was done after one cycle. A region with non-smooth sites was chosen for the ease of observation using IFM. Because the microscope adopts focus-variation principle, there needs to be sufficient contrast on the surface to obtain a meaningful measurement. As seen in Fig. 5, the surface textures remain identical before and after ion exchange. Their profile measurements based on surface metrology using IFM show that the shape and width of the selected area were retained after ion exchange. The error in the length of the bar is due to the difficulty of manually selecting the same profile at every observation for calculations.

Cs and Sr adsorption by the 3D-printed polymer matrix on its own was also tested as a control (Table S1†). From the XRF results, the polymer matrix itself does not exhibit any significant adsorption of either Cs or Sr. XRF data were collected for the pristine zeolite powders and two separate batches of 3D monoliths before and after exposure to Cs and Sr solutions (Tables 1 and 2). As absolute weight percentages vary depending upon many factors, the relative atomic ratios of elements, normalized to Al, have been calculated and are used for comparison. For all chabazite samples (Table 1), as expected, the Si/Al ratio remains the same at around 2.48 ± 0.15. For the chabazite samples before ion exchange, both Na and K are present as charge-balancing cations and the sum of their ratios to Al should equal to 1. The ratios are 1.06, 1.24 and 1.10 for the pristine powder, 3D-CHA monolith 1 and 3D-CHA monolith 2, respectively (Table 1). Given the fact that the data were collected on loose powders and many of the fluorescence lines are low energy, this is within acceptable error. After the Cs-exchange process, there is clear evidence of cesium uptake and loss of Na and K. The cation sums are 1.58, 2.39 and 2.83, respectively. These may be high due to an over-determination of the amount of cesium or indicate that in addition to the ion exchange uptake of the zeolite, a small amount of an insoluble cesium salt has formed. The determination of elements that fluoresce at higher energies, such as cesium *vs.* sodium, are often overestimated for thin samples as used here, as the thickness is insufficient for the assumption that all elements are present to an infinite thickness. The situation for Sr-exchange is identical, and as it is a divalent cation, the key sum is twice the Sr/Al ratio added to the other cation/Al ratios and these are 1.44, 1.54 and 2.01 for the pristine powder, 3D-CHA monolith 1 and 3D-CHA monolith 2, respectively. Critically, the 3D monoliths maintained their own shape after one day of shaking in solution, and no colloidal particles were observed after ion exchange. From Table 1, 3D-CHA behaves the same as the original form, and their Cs and Sr uptakes are proportional to the zeolite content. This also supports our claim that the low measured BET surface area was due to the low degassing temperature, and not due to blockage of the pores by the polymer. Based on these results, it can be concluded that the 3D-printing does not affect the ion exchange features in chabazite, and that its ion-capacity and performance are comparable to that of the zeolite only, but without the hazards associated with the powder form.

**Table 1:** Elemental composition of 3D-printed and powdered chabazite before and after ion exchange, analyzed using XRF

Element	Before ion exchange		Cs exchanged		Sr exchanged	
	wt%	Atomic ratio (normalised to Al)	wt%	Atomic ratio (normalised to Al)	wt%	Atomic ratio (normalised to Al)
**Powder (K, Na)-chabazite**						
Cs			17.84%	1.33		
Sr					8.92%	0.65
Si	18.33%	2.47	6.80%	2.39	8.22%	2.21
Al	7.12%	1	2.73%	1	3.87%	1
K	5.71%	0.55	1.01%	0.26	0.74%	0.13
Na	3.07%	0.51				
	Si/Al = 2.47		Si/Al = 2.39		Si/Al = 2.21	
	(K + Na)/Al = 1.06		(K + Cs)/Al = 1.58		(K + 2Sr)/Al = 1.44	

**3D-CHA monolith 1**						
Cs			24.67%	2.07		
Sr					6.79%	0.77
Si	6.62%	2.69	6.04%	2.40	6.21%	2.62
Al	2.36%	1	2.42%	1	2.49%	1
K	2.85%	0.83	1.14%	0.33	0.66%	0.18
Na	0.82%	0.41				
	Si/Al = 2.69		Si/Al = 2.40		Si/Al = 2.62	
	(K + Na)/Al = 1.24		(K + Cs)/Al = 2.39		(K + 2Sr)/Al = 1.54	

**3D-CHA monolith 2**						
Cs			24.07%	2.44		
Sr					6.53%	0.83
Si	7.86%	2.54	4.98%	2.39	6.03%	2.45
Al	2.97%	1	2.00%	1	2.22%	1
K	3.20%	0.74	1.12%	0.39	0.63%	0.18
Na	0.91%	0.36				
	Si/Al = 2.54		Si/Al = 2.39		Si/Al = 2.61	
	(K + Na)/Al = 1.10		(K + Cs)/Al = 2.83		(K + 2Sr)/Al = 2.01	

**Table 2:** Elemental composition of 3D-printed and powdered zeolite 4A before and after ion exchange, analyzed using XRF

Element	Before ion exchange		Cs exchanged		Sr exchanged	
	wt%	Atomic ratio (normalised to Al)	wt%	Atomic ratio (normalised to Al)	wt%	Atomic ratio (normalised to Al)
**Powder zeolite 4A**						
Cs			21.30%	1.05		
Sr					7.81%	0.61
Si	14.80%	1.08	4.49%	1.05	4.25%	1.0
Al	13.20%	1	4.12%	1	3.95%	1
Na	9.08%	0.81	2.31%	0.66	0.44%	0.13
	Si/Al = 1.08		Si/Al = 1.04		Si/Al = 1.03	
	Na/Al = 0.81		(Na + Cs)/Al = 1.71		(Na + 2Sr)/Al = 1.35	

**3D-4A monolith 1**						
Cs			22.41%	1.31		
Sr					5.87%	0.51
Si	9.67%	1.14	3.64%	1.01	4.15%	1.12
Al	8.14%	1	3.47%	1	3.55%	1
Na	5.68%	0.82	1.60%	0.54	0.81%	0.27
	Si/Al = 1.14		Si/Al = 1.01		Si/Al = 1.12	
	Na/Al = 0.82		(Na + Cs)/Al = 1.85		(Na + 2Sr)/Al = 1.29	

**3D-4A monolith 2**						
Cs			14.64%	0.76		
Sr					4.59%	0.38
Si	9.51%	1.17	4.46%	1.09	4.54%	1.18
Al	7.80%	1	3.92%	1	3.71%	1
Na	5.30%	0.80	2.13%	0.65	1.14%	0.36
	Si/Al = 1.17		Si/Al = 1.09		Si/Al = 1.18	
	Na/Al = 0.80		(Na + Cs)/Al = 1.40		(Na + 2Sr)/Al = 1.12	

**Fig. 6:**
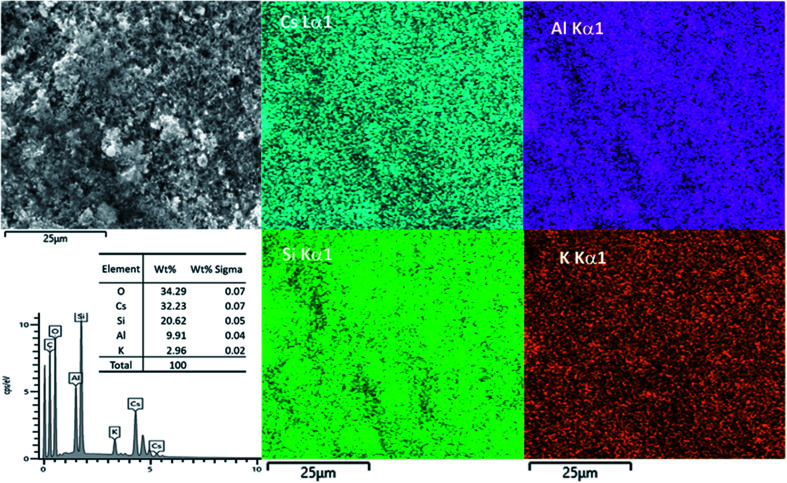
SEM image, EDX results and elemental mapping of Cs-exchanged 3D-CHA.

An attempt was made to use SEM-EDX-mapping to provide a better insight than XRF into the elemental distribution within the matrix. EDX maps of Cs-exchanged 3D-CHA are shown in Fig. 6. Unfortunately, chabazite is uniformly blended into the polymetric matrix and due to the very fine particle size, EDX-mapping could not distinguish regions of zeolite from polymetric matrix. The elemental composition of the Cs-exchanged CHA was measured and the Si/Al ratio from the average of five selected areas is 2.05 ± 0.05 and the (Na + K + Cs)/Al ratio is 0.99 ± 0.11, these are closer to the expected values than observed with XRF.

For all 4A samples, before and after ion exchange, the Si/Al ratio remains around 1.09 ± 0.06 (Table 2). For the zeolite 4A samples before ion exchange, Na is present as the charge-balancing cation and the sum of its ratio to Al should equal 1. It was noticed that cation/Al ratios were under-determined for samples before ion exchange and over-determined after Cs or Sr change. After the Cs and Sr-exchange process, there is clear evidence of Cs/Sr uptake and loss of Na. However, after one day of static ion exchange, none of the zeolite 4A samples was fully exchanged. Zeolite 4A exhibits better uptake towards smaller metal cations (Sr^2+^) than larger alkali metal cations (Cs^+^), and this phenomenon is consistent to all the powdered zeolite 4A or 3D-4A monoliths.

**Fig. 7:**
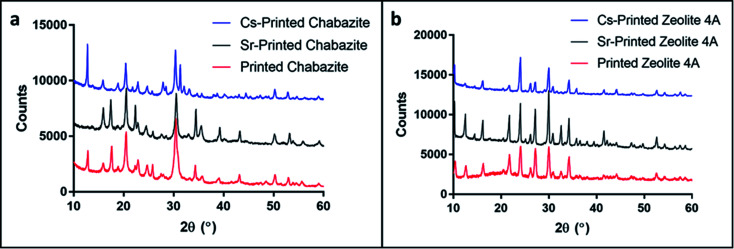
PXRD patterns of (a) 3D-CHA and (b) 3D-4A before and after ion exchange with Cs and Sr. The patterns of the printed systems have been offset for clarity.

An additional way to assess cation exchange in zeolites is to examine changes in the X-ray powder diffraction patterns. For 3D-CHA, the Cs- and Sr-exchanged samples remain with the same crystal structure and slightly bigger unit cells (Table 3 and Fig. S1†). Ion exchange typically results in a noticeable change in intensities due to heavy metal cations exchanging with light alkali cations inside the pores. As observed in Fig. 7a, the crystal structure of chabazite remained intact and noticeable changes in the relative intensities and unit cell dimensions were observed for both exchanged 3D materials. This further supports that ion exchange has occurred. Similarly, the crystal structure of zeolite 4A remained unchanged before and after ion exchange (Fig. 7b), and slight bigger unit cell dimensions for Cs and Sr-exchanged 4A were observed. The significant changes in peak intensities also further support the success of cation exchange.

**Table 3:** Unit cell parameters of the pre- and post-ion exchanged 3D-CHA and 3D-4A

	Crystal system	Space group	*a* [Å]	*b* [Å]	*c* [Å]	*V* [Å^3^]
3D-CHA	Rhombohedral	*R*3̄*m*	13.8321 (11)	13.8321 (11)	15.1547 (22)	2510.0 (6)
Cs-exchanged 3D-CHA	Rhombohedral	*R*3̄*m*	13.8680 (8)	13.8680 (8)	15.1188 (18)	2518.1 (4)
Sr-exchanged 3D-CHA	Rhombohedral	*R*3̄*m*	13.7826 (5)	13.7826 (5)	15.2636 (16)	2511.0 (3)
3D-4A	Cubic	*Pm*3̄*m*	12.2839 (7)	12.2839 (7)	12.2839 (7)	1853.6 (3)
Cs-exchanged 3D-4A	Cubic	*Pm*3̄*m*	12.3016 (4)	12.3016 (4)	12.3016 (4)	1861.6 (2)
Sr-exchanged 3D-4A	Cubic	*Pm*3̄*m*	12.3013 (3)	12.3013 (3)	12.3013 (3)	1861.4 (2)

**Fig. 8:**
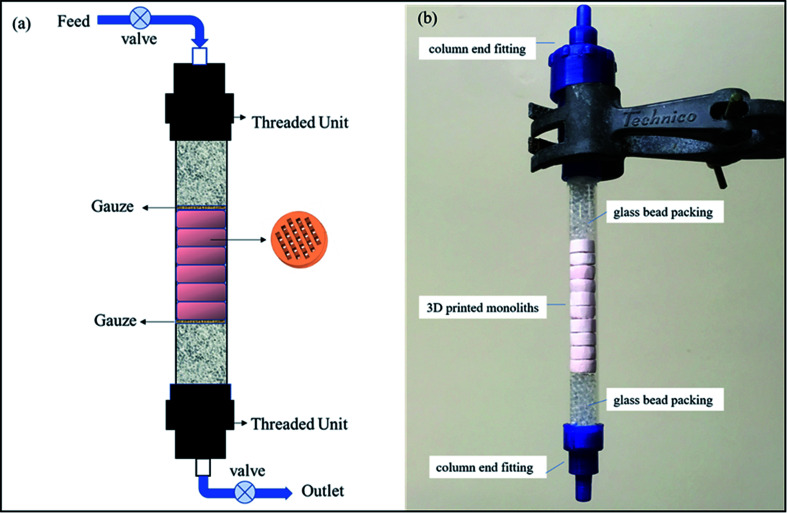
(a) Schematic diagram of an ion exchange column, (b) photograph of a packed column.

The most common uses of ion exchange media are as packed beds in vessels or columns. A stack of these 3D-printed monoliths can be packed in a customized small-volume column with an engineered inlet, outlet and flow distribution system to allow liquid to percolate through the bed of the medium at a specified flow rate. Fig. 8a represents a schematic diagram for a column set up, and a photograph of a mock column is presented in Fig. 8b. Retention screens on the inlet and outlet would prevent the medium from escaping into the process loop. After use, the robust monoliths could be readily removed and replaced in a simple operation. An additional advantage of the 3D-printing process is that the pore size and pore system within the monoliths can be tailored to suit the engineering flow requirements.

## 3. Conclusions

In summary, we have successfully demonstrated that 3D-printing enables the fabrication of porous hierarchical zeolite complex structures for utilization in nuclear wastewater decontamination. The monoliths retain the ion exchange properties of the zeolites and possess good mechanical stability. There is no reason to believe that the selectivities towards Cs^+^ and Sr^2+^ over other cations in solution will be different for the monoliths over zeolite powders. The DLP 3D-printing process demonstrated in this work enables control over the dimensions and shapes of the zeolite-embedded polymer, and over the degree of porosity and internal structure of the matrix. These features of the process enable its use for other separation processes, which are based on specific adsorption. In the case of nuclear waste treatment, in addition to the above-mentioned advantages, the printed columns enable simple and safe handling of the contaminated ion exchanger and may significantly reduce the risks and difficulties that rise when dealing with radioactive contaminated powders. We have not tested the radiological stability of the polymer matrix, but as the radioactive cations are trapped within the inorganic zeolite particles we would not expect any release of these into the environment even with polymer degradation. The polymer should also not significantly interfere with the thermal conversion of the spent exchangers into ceramic or vitreous wasteforms as it would be readily oxidized during the process without release of any radionuclides.

## 4. Experimental section

### 4.1 Synthesis of (K, Na)-chabazite

Chabazite was synthesised based on the method reported by the IZA commission.^43,44^ A 25 g portion of the ammonium form of zeolite Y (Alfa Aesar) (Powder, S.A. 750 m^2^ g^−1^, 5.2 : 1 mole ratio) was added into a solution made of water (198.2 mL) and KOH (26.8 mL, 45% solution). The mixture was sealed in a 500 mL polypropylene bottle and shaken for 30 s and then crystallised at 95 °C for 96 h. The product was filtered and thoroughly washed with DI-water. The (K, Na)-chabazite was prepared by repeated ion exchange of the as-synthesized K-chabazite with 1 M NaCl solutions.

### 4.2 Preparation of the printing formulation

The monomers, SR-508 (Sartomer) (1.8 g) and SR-349 (Sartomer) (4.2 g), were mixed with a dispersant, Anti-terra 203 (BYK) (0.8 g), and DB (Sigma) (12 g). The selected zeolite (6 g) was added and the mixture was stirred with a homogenizer (IKA T25) for 10 min. Then, the mixture was sonicated with a tip-sonicator (Sonics-Vibra cell, 500 W) for 10 min (1 s ON, 2 s OFF) at 40% amplitude. Following this, the pigment, Orasol orange 272 (BASF) (0.008 g), and the photoinitiator, Irgacure TPO (BASF) (0.0125 g) were added.

### 4.3 3D-printing and solvent exchange

The models were printed with a DLP 3D-printer (Asiga Pico 2). The printing parameters are presented in Table S2.† Once printed, the objects were washed with ethanol to remove any unpolymerized residues. Following this, the printed models were suspended in ethanol absolute (VWR) (20 mL) for 3 days to replace the DB. The ethanol was replaced with a new one every day to allow better removal of the DB.

### 4.4 Characterization of the printed models

The structural and morphological characterization of the printed models was performed with Powder X-Ray Diffraction (PXRD) (Shimadzu XRD-6000, Cu radiation), Scanning Electron Microscopy (Carl Zeiss SUPRA 55), Accelerated Surface Area and Porosimetry System (ASAP 2020, Micromeritics, degas at 70 °C) and Differential Scanning Calorimetry (Mettler-Toledo TGA/DSC 1 star system, sample is heated from 30–800 °C in air at the rate of 20 °C min^−1^). Pawley fits were performed using Total Pattern Solution (TOPAS 5) to calculate unit cell parameters before and after ion exchange.

### 4.5 Ion exchange test

The Cs and Sr uptake was tested individually by shaking the 3D-printed monoliths in 0.1 M Sr(NO_3_)_2_ or CsNO_3_ solution under batch conditions at v : m = 100 : 1 (mL : g) for 24 h at room temperature. The monoliths were rinsed with ∼50 mL of water and dried at 50 °C. The Cs and Sr-exchanged monoliths were grounded and analyzed in XRD (Bruker D8, Cu radiation) and XRF (Bruker S8 Tiger WDXRF, QUANT-EXPRESS software analysis). The Cs-exchanged monoliths were examined using SEM (FEI NOVA 200 Nano SEM) equipped with EDX and Alicona Infinite Focus Microscope.

## Conflicts of interest

There are no conflicts to declare.

## Supplementary Material

RA-010-C9RA09967K-s001

## References

[cit1] Pravalie R., Bandoc G. (2018). J. Environ. Manage..

[cit2] Kobayashi T., Nagai H., Chino M., Kawamura H. (2013). J. Nucl. Sci. Technol..

[cit3] Dyer A., Hriljac J., Evans N., Stokes I., Rand P., Kellet S., Harjula R., Moller T., Maher Z., Heatlie-Branson R., Austin J., Williamson-Owens S., Higgins-Bos M., Smith K., O'Brien L., Smith N., Bryan N. (2018). J. Radioanal. Nucl. Chem..

[cit4] Mimura H., Kanno T. (1985). J. Nucl. Sci. Technol..

[cit5] Baek W., Ha S., Hong S., Kim S., Kim Y. (2018). Microporous Mesoporous Mater..

[cit6] Dyer A., Zubair M. (1998). Microporous Mesoporous Mater..

[cit7] Schmidt W. , in Handbook of Porous Solids, ed. F. Schuth, K. S. W. Sing and J. Weitkamp, Wiley-VCH, 2008, 10.1002/9783527618286.ch18g

[cit8] Munthali M. W., Johan E., Aono H., Matsue N. (2015). J. Asian Ceram. Soc..

[cit9] Anthony R. G., Dosch R. G., Gu D., Philip C. V. (1994). Ind. Eng. Chem. Res..

[cit10] Huckman M. E., Latheef I. M., Anthony R. G. (1999). Sep. Sci. Technol..

[cit11] Clearfield A., Bortun A. I., Bortun L. N., Poojary D. M., Khainakov S. A. (1998). J. Mol. Struct..

[cit12] Möller T., Clearfield A., Harjula R. (2002). Microporous Mesoporous Mater..

[cit13] Liu B. J., Mu W. J., Xie X., Li X. L., Wei H. Y., Tan Z. Y., Jian Y., Luo S. Z. (2015). RSC Adv..

[cit14] Fewox C. S., Clearfield A., Celestian A. J. (2011). Inorg. Chem..

[cit15] Sylvester P. , in Encyclopedia of Separation Science, ed. I. D. Wilson, Academic Press, Oxford, 2000, pp. 4261–4267

[cit16] Nyman M. , Nenoff T. M. and Headley T. J., Characterization of UOP IONSIV IE911, 2001

[cit17] Miller J. E. , Brown N. E., Krumhansl J. L., Trudell D. E., Anthony R. G. and Philip C. V., in Science and Technology for Disposal of Radioactive Tank Wastes, ed. W. W. Schulz and N. J. Lombardo, Springer US, Boston, MA, 1998, pp. 269–286

[cit18] Ngo T. D., Kashani A., Imbalzano G., Nguyen K. T. Q., Hui D. (2018). Composites, Part B.

[cit19] Panda B., Tay Y. W. D., Paul S. C., Tan M. J. (2018). Materialwiss. Werkstofftech..

[cit20] Erkal J. L., Selimovic A., Gross B. C., Lockwood S. Y., Walton E. L., McNamara S., Martin R. S., Spence D. M. (2014). Lab Chip.

[cit21] Haghiashtiani G., Habtour E., Park S. H., Gardea F., McAlpine M. C. (2018). Extreme Mech. Lett..

[cit22] Gnanasekaran K., Heijmans T., van Bennekom S., Woldhuis H., Wijnia S., de With G., Friedrich H. (2017). Appl. Mater. Today.

[cit23] Kim K., Zhu W., Qu X., Aaronson C., McCall W. R., Chen S. C., Sirbuly D. J. (2014). ACS Nano.

[cit24] Halevi O., Tan J. M. R., Lee P. S., Magdassi S. (2018). Adv. Sustainable Syst..

[cit25] Regufe M. J., Ferreira A. F. P., Loureiro J. M., Rodrigues A., Ribeiro A. M. (2019). Microporous Mesoporous Mater..

[cit26] Couck S., Cousin-Saint-Remi J., Van der Perre S., Baron G. V., Minas C., Ruch P., Denayer J. F. M. (2018). Microporous Mesoporous Mater..

[cit27] Li Y. H., Chen S. J., Cai X. H., Hong J. Q., Wu X., Xu Y. Z., Zou J. J., Chen B. H. (2018). J. Mater. Chem. A.

[cit28] Thakkar H., Eastman S., Hajari A., Rownaghi A. A., Knox J. C., Rezaei F. (2016). ACS Appl. Mater. Interfaces.

[cit29] Li X., Li W. B., Rezaei F., Rownaghi A. (2018). Chem. Eng. J..

[cit30] Fu S., He P., Wang M., Wang M., Wang R., Yuan J., Jia D., Cui J. (2019). J. Eur. Ceram. Soc..

[cit31] Lefevere J., Protasova L., Mullens S., Meynena V. (2017). Mater. Des..

[cit32] Truby R. L., Lewis J. A. (2016). Nature.

[cit33] I. A. E. Agency , Application of Ion Exchange Processes for Treatment of Radioactive Waste and Management of Spent Ion Exchangers, International Atomic Energy Agency, Vienna, 2002

[cit34] Klika Z., Kraus L., Vopalka D. (2007). Langmuir.

[cit35] Liu H. J., Xie S. B., Xia L. S., Tang Q., Kang X., Huang F. (2016). Environ. Earth Sci..

[cit36] Plecas I., Dimovic S., Smiciklas I. (2006). Prog. Nucl. Energy.

[cit37] Kuang X., Chen K. J., Dunn C. K., Wu J. T., Li V. C. F., Qi H. J. (2018). ACS Appl. Mater. Interfaces.

[cit38] Han D., Lu Z. C., Chester S. A., Lee H. (2018). Sci. Rep..

[cit39] Li Y. Y., Tolley H. D., Lee M. L. (2010). J. Chromatogr. A.

[cit40] Li Y. Y., Tolley H. D., Lee M. L. (2011). J. Chromatogr. A.

[cit41] Viklund C., Svec F., Frechet J. M. J., Irgum K. (1996). Chem. Mater..

[cit42] Santora B. P., Gagne M. R., Moloy K. G., Radu N. S. (2001). Macromolecules.

[cit43] Bourgogne M. , Guth J.-L and Wey R., *US Pat.*, 4 503 024, 1985

[cit44] Verified Syntheses of Zeolitic Materials, ed. H. Robson and K. P. Lillerud, Elsevier Science, Amsterdam, 2001, pp. 123–125, 10.1016/B978-044450703-7/50132-0

