# Supplementary material for: Nuclear wastewater decontamination by 3D-Printed hierarchical zeolite monoliths
Source: RSC Adv. 2020 Feb 5;10(10):5766–76. doi: 10.1039/c9ra09967k (PMC9049225; doi:10.1039/c9ra09967k)
Supplement: RA-010-C9RA09967K-s001 [file RA-010-C9RA09967K-s001.pdf]

Electronic Supplementary Material (ESI) for RSC Advances.
This journal is © The Royal Society of Chemistry 2020

Supporting Information

Nuclear Wastewater Decontamination by 3D-Printed Hierarchical Zeolite Monoliths

Oded Halevi, Tzu-Yu Chen, Pooi See Lee, Shlomo Magdassi*, Joseph A. Hriljac**

O. H. and T.-Y. C. contributed equally to this work.

Dr. J. A. Hriljac, Dr. T.-Y. Chen
School of Chemistry, University of Birmingham, Edgbaston, Birmingham, B15 2TT, UK.
Dr. J. A. Hriljac
Diamond Light Source Ltd, Harwell Science and Innovation Campus, Didcot, OX11 0DE, UK.
Dr. T.-Y. Chen
Materials and Engineering Research Institute, Faculty of Science, Technology and Arts, Sheffield Hallam University, City Campus, Howard Street, Sheffield, S1 1WB, UK
Prof. S. Magdassi, Prof. P. S. Lee, O. Halevi
CREATE NTU-HUJ Programme, Enterprise Wing, 138602, Singapore.
Prof. S. Magdassi, O. Halevi
Casali Center for Applied Chemistry, Institute of Chemistry, The Hebrew University of Jerusalem, Jerusalem, 91904, Israel.
Prof. P. S. Lee, O. Halevi
School of Materials Science and Engineering, Nanyang Technological University, 639798, Singapore.
E-mail: joseph.hriljac@diamond.ac.uk; magdassi@mail.huji.ac.il; pslee@ntu.edu.sg

1. Table S1. XRF results of Cs and Sr adsorption on 3D printed polymer metrices
2. Table S2. Printing parameters
3. Figure S1. Pawley fits of 3D-CHA and 3D-zeolite 4A before and after Cs and Sr exchanges

Table S1. XRF results of Cs and Sr adsorption on 3D printed polymer metrices

	**control sample 1**		**control sample 2**		**control sample 3**	
**Cs adsorption**	Formula	wt%	Formula	wt%	Formula	wt%
	Ce	0.11%	P	0.09%	Ce	0.11%
	P	0.10%	Cr	0.03%	P	0.06%
	Ca	0.06%	Fe	0.02%	Fe	0.02%
	Cl	0.06%			Si	0.02%
	Zr	0.04%			S	0.01%
	Fe	0.04%			Mg	0.01%
	S	0.03%				
	Mg	0.02%				
	V	0.02%				
**Sr adsorption**	Formula	wt%	Formula	wt%	Formula	wt%
	P	0.11%	P	0.03%	P	0.11%
	Al	0.09%	Fe	0.03%	Na	0.06%
	Ca	0.05%	Mo	0.02%	Fe	0.04%
	Sr	0.05%	Sr	0.02%	Cl	0.03%
	Cr	0.04%	Mg	0.01%	Ca	0.03%
	Cu	0.02%			Si	0.03%
	Ti	0.02%				

Table S2. Printing parameters

Parameter	Value
Light Intensity	39.158 [mW/cm^2]
Slice thickness	0.050 [mm]
Burn-In Exposure Time	45.000 [s]
Exposure Time	12.000 [s]
Separation Velocity	1.000 [mm/s]
Separation Distance	5.000 [mm]
Approach Velocity	2.000 [mm/s]
Slides Per Layer	1.000
Slide Velocity	10.000 [mm/s]
Burn-In Wait Time (After Exposure)	5.000 [s]
Burn-In Wait Time (After Separation)	5.000 [s]
Burn-In Wait Time (After Approach)	0.000 [s]
Burn-In Wait Time (After Slide)	0.000 [s]
Normal Wait Time (After Exposure)	5.000 [s]
Normal Wait Time (After Separation)	3.000 [s]
Normal Wait Time (After Approach)	0.000 [s]
Normal Wait Time (After Slide)	0.000 [s]

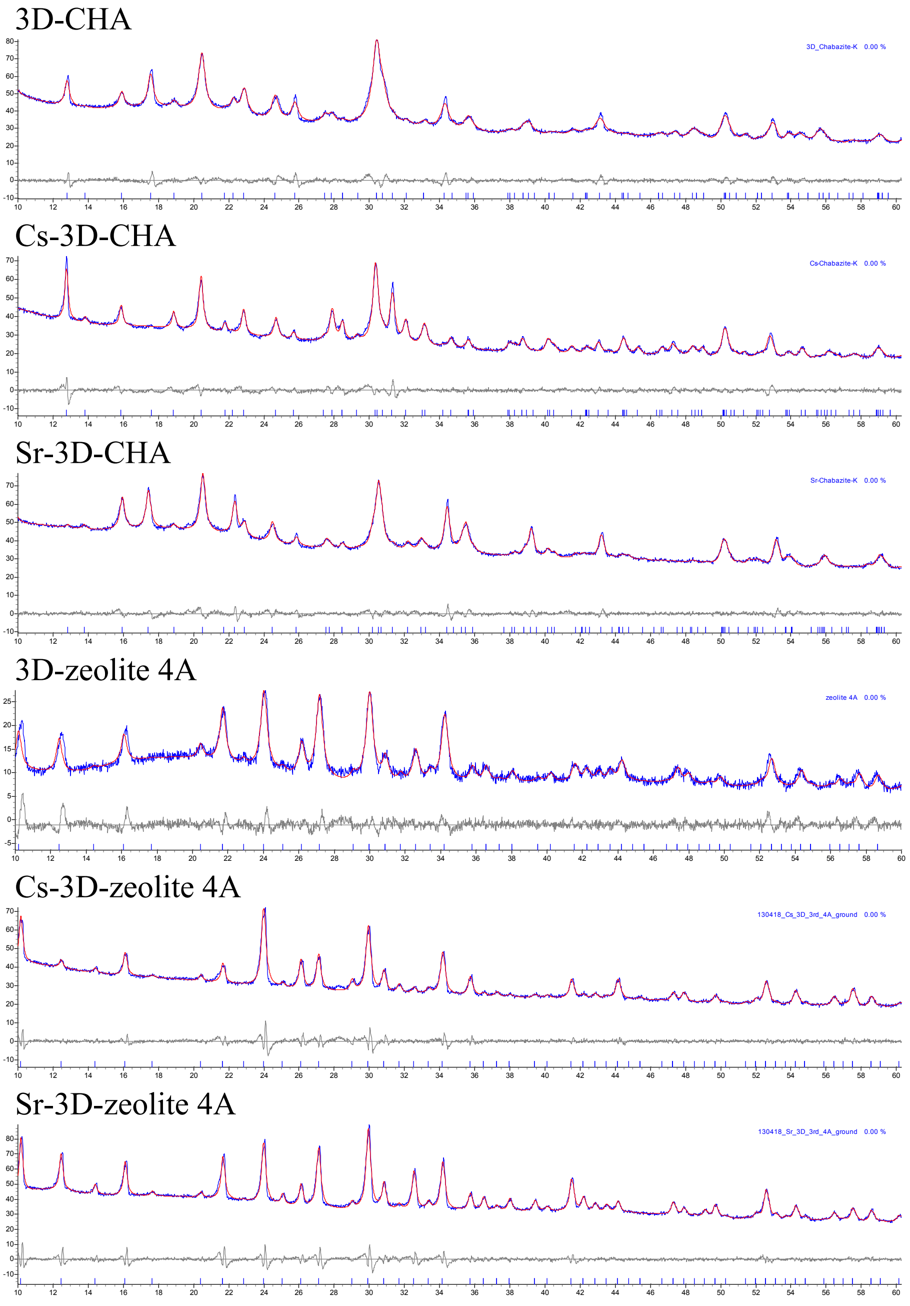

Figure S1. Pawley fits of 3D-CHA and 3D-zeolite 4A before and after Cs and Sr exchanges.
